# Analysis of Human Papillomavirus (HPV) 16 Variants Associated with Cervical Infection in Italian Women

**DOI:** 10.3390/ijerph17010306

**Published:** 2020-01-01

**Authors:** Marianna Martinelli, Chiara Villa, Giovanni Sotgiu, Narcisa Muresu, Federica Perdoni, Rosario Musumeci, Romina Combi, Antonio Cossu, Andrea Piana, Clementina Cocuzza

**Affiliations:** 1 Department of Medicine and Surgery, University of Milano-Bicocca, 20900 Monza, Italy; chiara.villa@unimib.it (C.V.); federica.perdoni@unimib.it (F.P.); rosario.musumeci@unimib.it (R.M.); romina.combi@unimib.it (R.C.); clementina.cocuzza@unimib.it (C.C.); 2 Department of Medical, Surgical and Experimental Sciences, University of Sassari, 07100 Sassari, Italy; gsotgiu@uniss.it (G.S.); narcisamuresu@outlook.com (N.M.); antonio.cossu@aousassari.it (A.C.); piana@uniss.it (A.P.)

**Keywords:** HPV genotypes, HPV variants, LCR mutations, HPV16

## Abstract

This study aims to evaluate HPV16 variants distribution in a population of Italian women living in two different regions (Lombardy and Sardinia) by sequence analyses of HPV16-positive cervical samples, in order to reconstruct the phylogenetic relationship among variants to identify the currently circulating lineages. Analyses were conducted starting from DNA isolated from 67 HPV16-positive cervical samples collected from two different Italian centres (31 from Lombardy and 36 from Sardinia) of women with normal and abnormal cervical cytology. The entire long control region (LCR) and 300 nt of the *E6* gene was sequenced to identify intra-type variants. Sequence comparison and phylogenetic analysis were made using a distance-based neighbour joining method (NJ) and Kimura two-parameter model. Data obtained reported that Italian sequences mainly belonged to the European lineage, in particular sublineage A2. Only five sequences clustered in non-European branches: two in North American lineage (sublineage D1), two in African-1 (sublineage B1) and one in African-2. A new 27 nucleotide duplication in the central segment of the LCR region was found in a sequence obtained from a sample isolated in Sardinia. A predominance of European variants was detected, with some degree of variability among the studied HPV16 strains. This study contributes to the implementation of data regarding the molecular epidemiology of HPV16 variants.

## 1. Introduction

Human papillomavirus (HPV), recognized as the etiological agent of genital and extra-genital cancers, is a highly conserved circular double-stranded DNA virus [1]. Its genome size consists of ~8000 bases in length and is divided into three functional regions: a non-coding upstream regulatory region (URR), also known as the long control region (LCR), containing regulatory elements for viral replication and transcription; an early region (*E1*, *E2*, *E4*–*E7* genes) encoding core viral proteins (e.g., E6 and E7 proteins can cause transformation of the host cell); a late region, encoding L1 and L2 capsid proteins responsible for the viral DNA packaging and assembling in an icosahedral structure [2].

Currently, >200 HPV types have been identified based on >10% nucleotide diversity of *L1* gene [3,4]. The International Agency for Research on Cancer (IARC) classified twelve HPV genotypes as “high-risk” (hrHPV) based on their association with cancers: 16, 18, 31, 33, 35, 39, 45, 51, 52, 56, 58, and 59 [5]. Since HPV genome replication relies on high-fidelity DNA polymerases of the host cell with proofreading activity, the high genetic diversity of HPV strains is presumably the result of a long evolutionary history with the sequential accumulation of genetic changes through intracellular mutagenic processes [6]. 

Epidemiological and biological studies found that HPV16 is the most frequently detected HPV genotype in patients with cervical cancer worldwide [6]. Moreover, it is also the predominant infection identified among subjects with or without genital lesions in different groups of the population [7,8,9,10].

Based on sequence diversity, HPV16 has been grouped into four phylogenetic lineages: A, B, C and D. A distinct lineage differs by between 1.0% and 10% at the whole-genome nucleotide level, and is further divided into sublineages if the nucleotide difference between two variants from the same lineage is between 0.5% and 1.0% [11]. Lineage A comprises four sublineages: A1, A2, A3 (includes European sequences worldwide) and A4 (Asian sequences). Lineage B is further divided into sublineages B1 and B2, which includes African sequences, similarly to lineage C. Lineage D in turn is subclassified into D1, D2 and D3, and comprises Asian American and North American sequences isolated worldwide [12]. The distribution of HPV16 variants depends on geography, ethnicity, and carcinogenicity [13]. The European HPV16 A1, A2 and A3 sublineages account for the majority of HPV16 infections worldwide [14,15], whereas the frequency of non-European B, C, and D variants is associated with an increased risk of cancer progression [16] and severity of cervical lesions [17,18]. The knowledge of HPV16 variants circulating in a specific geographical region might be important for cervical cancer prevention. The aim of the study was to investigate the HPV16 variant distribution in cervical samples collected from women living in two different Italian regions (i.e., Lombardy and Sardinia) and to assess the phylogenetic relationships among variants.

## 2. Materials and Methods

### 2.1. DNA Samples

This study was conducted by analyzing DNA isolated from HPV16-positive cervical samples of women with normal and abnormal cytology. In particular, women attending two gynecology clinics (one in Lombardy, Northern Italy, and the other in Sardinia, Southern Italy) between January and June 2018 for routine investigations were enrolled. Sixty-seven samples found to be positive for HPV16 were further analysed. All subjects provided written informed consent to participate in the study. The study protocol was approved by the University of Milano-Bicocca, Monza, Italy (Protocol; n. 305).

DNA extraction was performed using NucliSENS^®^ easyMag^®^ (bioMérieux, Marcy-l’Étoile, France) system. HPV16 positivity was confirmed by the Anyplex™II HPV28, (Seegene, Seoul, Korea). Cervical cytological alterations were classified according to the 2001 Bethesda System [19].

### 2.2. PCR Amplification and Sequencing

HPV DNA amplification was performed in a 50 μL reaction mix containing GoTaq^®^ Flexi DNA Polymerase (Promega, Madison, WI, USA), 0.75 μM of each primer (16-F101 and 16-R20) [20] and 5 μL of the template sample. Thermocycling conditions used were: 95 °C for 5 min followed by 40 cycles of 98 °C for 30 s, 63 °C for 30 s, 72 °C for 2 min, and final elongation at 72 °C for 5 min.

Amplicons were visualized on 2% agarose gel stained with Atlas ClearSight DNA Stain (Bioatlas, Tartu, Estonia). Nucleotide sequences were obtained through the Sanger sequencing method (BigDye Terminator Cycle Sequencing kit v1.1) and automated ABI PRISM 3130 genetic analyzer (ThermoFisher, Waltham, MA, USA). The resulting sequence fragment corresponds to a 1160 bp stretch covering the entire LCR and the 300 nt of the E6 ORF.

### 2.3. Phylogenetic and Statistical Analyses

Sequences were aligned using ClustalW software, followed by manual editing using BioEdit software (available at http://www.mbio.ncsu.edu/bioedit/bioedit.html) [21]. Phylogenetic analysis was performed using a distance-based neighbour joining method (NJ) and Kimura two-parameter model implemented in the MEGA version 7 program [22] (version 7.0.14, available at http://www.megasoftware.net/). The reliability of the observed clades was proved with internal node bootstrap values ≥70% (after 1000 replicates). Reference sequences were obtained from Papilloma Episteme (PaVE, https://pave.niaid.nih.gov).

All sequences were submitted to the NCBI database and their accession numbers in GenBank are from MN848167 to MN848233.

## 3. Results

### 3.1. Study Population

The median interquartile range (IQR) age of the study population was 36 (29–46) years. Twenty-seven (27/31; 87.1%) and thirty-one (31/36, 86.1%) of the women enrolled in Lombardy and Sardinia had an abnormal cervical cytology result, respectively (Table 1).

### 3.2. Phylogenetic Reconstruction

**Figure 1:**
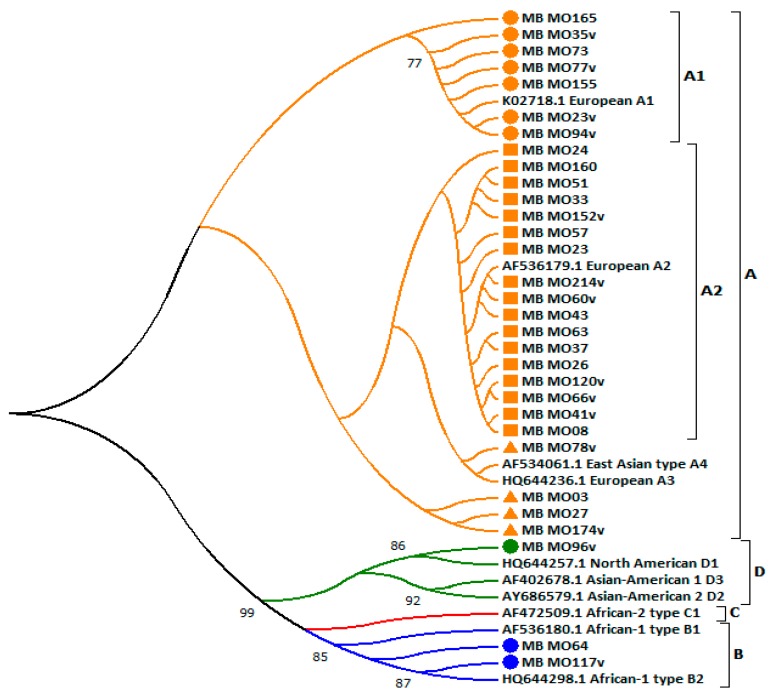
Evolutionary relationships of HPV16 sequences isolated from 31 women in the centre located in Lombardy. Sequences that belong to lineage A are highlighted in orange: the circles identify sequences clustered to sublineage A1, rectangles sequences clustered to sublineage A2 and triangles sequences clustered to an unclear A sublineage, respectively. Sequences that cluster to lineages B, C and D are reported in blue, red and green, respectively. Of these, sequences isolated from women enrolled in this centre are marked with a circle. The evolutionary history was inferred using the Neighbor-Joining method. The optimal tree with the sum of branch length = 0.08674820 is shown. The evolutionary distances were computed using the Kimura two-parameter method. The analysis involved 41 nucleotide sequences. Evolutionary analyses were conducted in MEGA7.

Phylogenetic analysis of 31 strains isolated in Lombardy and 10 reference sequences showed that 90.1% (30/33) belonged to the A lineage (Figure 1). In particular, the majority (17/33, 51.5%) were grouped in the A2 sublineage and clustered with the sequence AF536179, whereas seven isolates clustered in the A1 sublineage (K02718), and four were grouped into the A lineage but without a clear sublineage. Non-European variants accounted for three (9.7%) infections: one was included in the D lineage (within D1 sublineage) and two clustered within the B lineage (within B1 sublineage).

Ten out of 28 (35.7%) European variants were collected from patients with low-grade squamous intraepithelial lesion (LSIL), seven (25.0%) from high-grade squamous intraepithelial lesion (HSIL), five (17.8%) from atypical squamous cells of undetermined significance (ASCUS), two (2.5%) from atypical squamous cells (cannot exclude HSIL) (ASCH), and the remaining in negative for intraepithelial lesion or malignancy (NILM) smears. The three variants belonging to lineages B and D were collected from three women: one with HSIL, one with LSIL, and one with NILM cervical cytological positive samples (Table 2).

**Figure 2:**
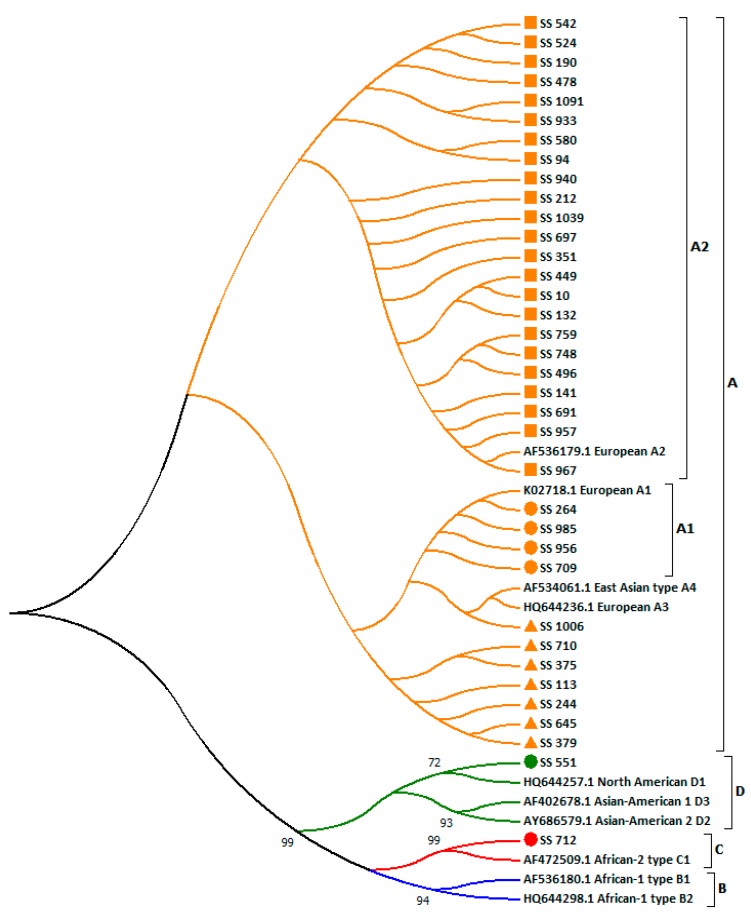
Evolutionary relationships of HPV16 sequences isolated from 36 women in the centre located in Sardinia. Sequences that belong to lineage A are highlighted in orange: circles identify sequences clustered to sublineage A1, rectangles sequences clustered to sublineage A2 and triangles sequences clustered to an unclear A sublineage, respectively. Sequences that cluster to lineages B, C and D are reported in blue, red and green, respectively. Of these, sequences isolated from women enrolled in this centre are marked with a circle. The evolutionary history was inferred using the Neighbor-Joining method. The optimal tree with the sum of branch length = 0.08407424 is shown. The evolutionary distances were computed using the Kimura two-parameter method. The analysis involved 46 nucleotide sequences. Evolutionary analyses were conducted in MEGA7.

Phylogenetic analysis of the 36 strains isolated in Sardinia and 10 reference sequences showed that the majority (34/36; 94.4%) of the HPV16 strains belonged to the A lineage (Figure 2). In particular, 23 (63.9%) were grouped into the A2 sublineage, four into the A1 sublineage, and seven into the A lineage but with unclear sublineage. Two (5.6%) were included in Non-European clusters, one clustered into the D lineage (D1 sublineage), and one within the C lineage.

Five out of 34 (14.7%) European variants were obtained from patients with HSIL, one from ASCH, 24 from ASCUS, and the remaining ones were obtained from NILM. Two samples belonging to Non-European variants were isolated from women with ASCUS (Table 2).

### 3.3. LCR Nucleotide Sequences Analysis

All HPV16 sequences were compared with the prototype clone (K02718), analysing the LCR region. A total of 40 single nucleotide polymorphisms (SNPs) were detected (Table 3). The majority of sequences showed the presence of more than one SNP (62/67, 92.5%). Three SNPs were the most frequently detected: C7434G (67/67, 100%), G7519A (57/67, 85%) and G7190T (55/67, 82%). The last two SNPs were not found among all sequences clustered into the A1 sublineage.

**Figure 3:**
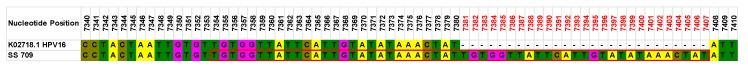
Alignment of partial SS 709 LCR sequence (7340–7410 nt) with K02718 HPV16 reference sequence. The 27 nt duplication detected is highlighted in red.

A very interesting mutation was found in the SS 709 sequence isolated in Sardinia, showing the duplication of 27 nucleotides at position 7381 of the HPV genome (Figure 3). This mutation was detected in a sample collected from a woman that reported ASCUS as a result of cytological examination. This woman was referred to colposcopy for further investigation and the biopsy result showed the presence of CIN2-grade cervical dysplasia. This sample was retested to confirm the presence of this duplication.

## 4. Discussion

Phylogenetic analysis of LCR-E6 sequences showed that almost all HPV16 variants belonged to the A lineage. In particular, the most prevalent sublineage in the studied Italian HPV16 positive cervical samples was found to be A2. Several Italian studies were performed in human immunodeficiency virus (HIV)-positive or -negative [23,24,25,26], Caucasian [27,28] and non-Caucasian women [29]. Overall, previous data showed that most of the HPV16 isolates circulating in Italy belong to the A lineages [28,29], while the B and D lineages have been reported in women with high-grade cervical lesions and cancer [28,29,30]. Lineage C was detected in high-risk women immigrants (West Africa) living in Southern Italy and HIV-positive subjects [24,25,27]. A recent study also showed the circulation of A2, B2, B4, C1, and D4 HPV16 sublineages among immigrant women in Calabria region, potentially caused by its geographical position in the Mediterranean area [27]. On the contrary, in this study we also observed the presence of Non-European variants (B1, C1 and D1) among Italian women without cervical cancer but with an ongoing or regressed cervical lesions. Moreover, to our knowledge, this is the first study that analyses HPV16 variants distribution in samples collected from women who attended a gynecology centre in Sardinia. Interestingly, although Lombardy and Sardinia are characterized by different geographical, demographic, and historical factors, the only difference detected in the samples collected from these two centres was found in the lineages of the Non-European variants (B in Lombardy and C in Sardinia). The relatively small number of sequences studied and the low detection rate of Non-European variants cannot prove any statistical relationship between Pap smear results and HPV16 variants. The LCR, which is the most variable region of the HPV genome, plays an important role in viral transcription and replication, as well as the persistence of the viral infection and risk of progression to cervical cancer [31]. Different nucleotide mutations in the LCR have been reported, some of them related to altered pathways involved in viral persistence and cancer development [31]. A Chinese study described two LCR nucleotide mutations (G7193T and G7518A) which were the potential binding sites of FOXA1 (Forkhead box protein A1) and SOX9 (sex-determining region Y-box 9) transcriptional factors, respectively [32]. To the best of our knowledge, a newly described duplication of 27 nucleotide at position 7381, located in the 5′ segment of the LCR region [33,34], was identified in our study. The biological and clinical role of the duplication demonstrated in this study will be further evaluated. However, considering the results obtained by Xi and colleagues, this duplication does not fall into the LCR region and should not influence the binding sites of transcription factors [32]. Carson et al. also reported no specific transcriptional binding sites inside or next to the LCR region where we identified the 27 nt duplication [35]. Previous studies reported the presence of other different duplications in the HPV16 and in other HPV genotypes’ genomes. For example, Choo et al. showed the presence of a 1330 bp sequence duplication containing the long control region (LCR) and the E6 and E7 ORFs in an HPV16 genome cloned from a cervical carcinoma [36]. A 213 base pairs duplication mapped in the LCR was found in an HPV16 sample isolated from vulvar Bowenoid papulosis. This variant maps upstream of a region containing several regulatory elements [37]. Regarding the other HPV genotypes, in a study conducted in the USA, DNA analysis revealed a duplication of 2493 bp that includes a partial L1-long control region (LCR)–E6–E7-partial E1 sequences in an HPV-11 genome isolated from a lung lesion of a patient with recurrent respiratory papillomatosis. These data suggest a link between the duplication of the HPV-11 promoter and *E6/E7* oncogenes and the clinical aggressiveness of the tumor in recurrent respiratory papillomatosis [38]. In another study, a 170 base pair duplication was identified within the LCR of HPV6 from a patient with aggressive recurrent respiratory papillomatosis, suggesting a possible influence of promoter activity [39].

Forty SNPs in the LCR were detected in our studied samples, and C7434G, G7519A and G7190T were the most commonly detected. A very high percentage of G7519A and G7190T detection was also shown in other studies conducted in England, Poland and Mexico [20,40,41]. Both mutations are located within YY1 transcriptional factor binding sites that have been reported to be involved in the regulation of HPV16 E6/E7 oncogenic transcripts [42]. Therefore, mutations within or close to these sites could alter viral oncogenic potential. Other mutations, such as A7231C, G7700A, A7727C and G7866A, detected with lower percentages, have already been reported in a recent study conducted by Zhe and et al. [43].

A more comprehensive overview of HPV variants circulating in Italy is needed, investigating more regions, to better assess the key genomic differences of HPV variants and their involvement in viral persistence and tumor progression.

## 5. Conclusions

European variants were highly prevalent in our Italian samples. Although most of the sequences reported in this study have been previously described, a new duplication in LCR has been detected. These findings could help to better understand the molecular epidemiology of HPV16 variants. It will be important to follow-up our findings in subsequent studies to characterize genetic variations among HPV16 genome in more depth. A priority should be to conduct clinical and functional studies to understand how specific mutations can be linked to persisting infections and different clinical outcomes.

## Figures and Tables

**Table 1:** Pap test results of the human papillomavirus (HPV)16-positive women enrolled. High-grade squamous intraepithelial lesion (HSIL), atypical squamous cells—cannot exclude HSIL (ASCH), low-grade squamous intraepithelial lesion (LSIL), atypical squamous cells of undetermined significance (ASCUS), and negative for intraepithelial lesion or malignancy (NILM).

*Cytology*	*HSIL n. (%)*	*ASCH n. (%)*	*LSIL n. (%)*	*ASCUS n. (%)*	*NILM n. (%)*
***Lombardy Centre (n = 31)***	8 (25.8%)	2 (6.5%)	10 (32.2%)	7 (22.6%)	4 (12.9%)
***Sardinia Centre (n = 36)***	5 (13.9%)	1 (2.8%)	0 (0.0%)	25 (69.4%)	5 (13.9%)

**Table 2:** HPV16 lineage distribution by cytological result.

*HPV16 Lineage*	*Cytology*				
	HSIL	ASCH	LSIL	*ASCUS*	*NILM*
***Lombardy Centre (n = 31)***					
***A (n = 28)***	7	2	10	5	4
***B (n = 2)***	0	0	1	0	1
***D (n = 1)***	1	0	0	0	0
***Sardinia Centre (n = 36)***					
***A (n = 34)***	5	1	0	24	4
***C (n = 1)***	0	0	0	1	0
***D (n = 1)***	0	0	0	1	0

**Table 3:** Nucleotide sequence variations in the LCR of HPV 16 (*n* = 67).

Nucleotide Position	Nucleotide Change	*n*. (%)
7171	G > C	2 (2.99)
7173	A > C	1 (1.49)
**7190**	**G > T**	**55 (82.09)**
7196	T > A	3 (4.48)
7225	A > C	2 (2.99)
7230	A > G	1 (1.49)
	A > C	1 (1.49)
7231	A > C	3 (4.48)
7281	T > G	1 (1.49)
7314	A > C	3 (4.48)
7346	A > C	1 (1.49)
7372	A > G	1 (1.49)
7374	A > T	1 (1.49)
7413	C > T	1 (1.49)
7433	G > A	2 (2.99)
**7434**	**C > G**	**67 (100.00)**
7445	G > A	1 (1.49)
7448	T > C	11 (16.42)
7470	A > T	1 (1.49)
7483	A > C	3 (4.48)
7487	G > A	6 (8.96)
7494	T > C	6 (8.96)
**7519**	**G > A**	**57 (85.07)**
7620	T > C	1 (1.49)
7653	C > G	1 (1.49)
7667	C > T	4 (5.98)
7687	C > A	5 (7.46)
7700	G > A	1 (1.49)
7727	A > C	3 (4.48)
7728	A > C	1 (1.49)
7762	C > T	6 (8.96)
7766	G > A	1 (1.49)
7784	C > T	5 (7.46)
7790	C > A	1 (1.49)
7797	G > C	1 (1.49)
7824	G > A	2 (2.99)
7832	G > T	5 (7.46)
7835	A > C	1 (1.49)
7837	A > G	1 (1.49)
7866	G > A	4 (5.98)
